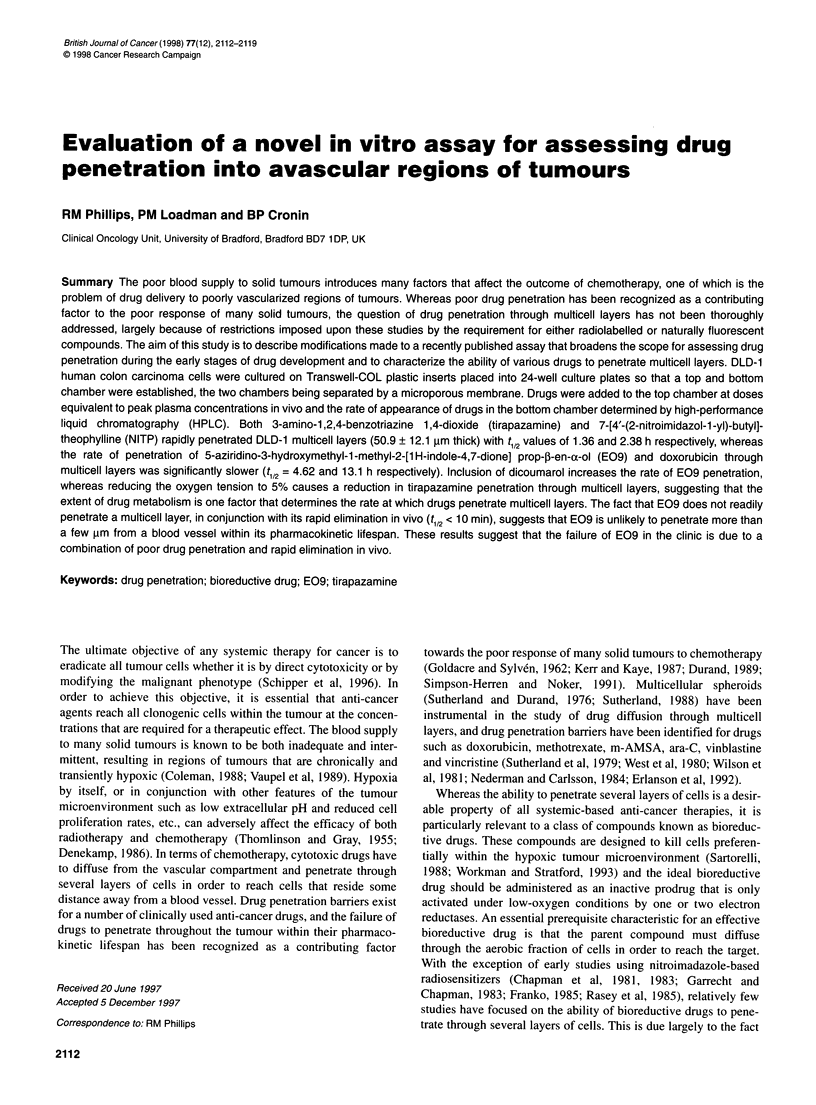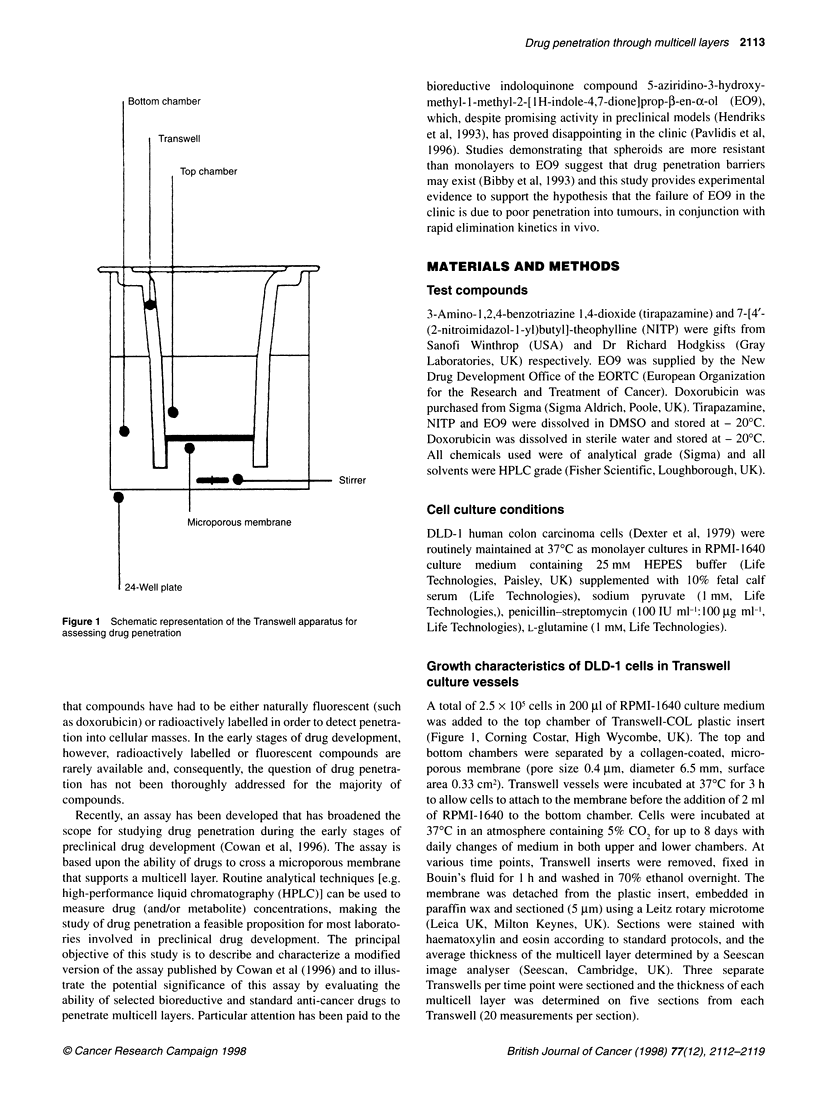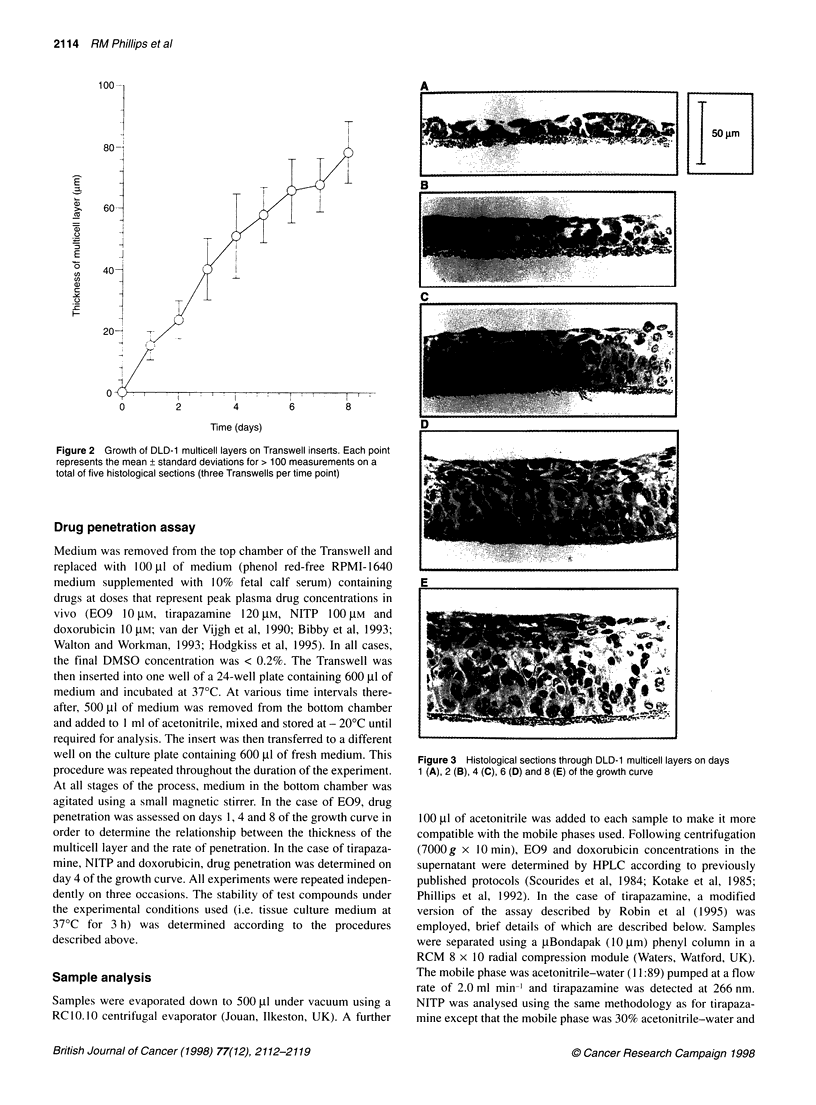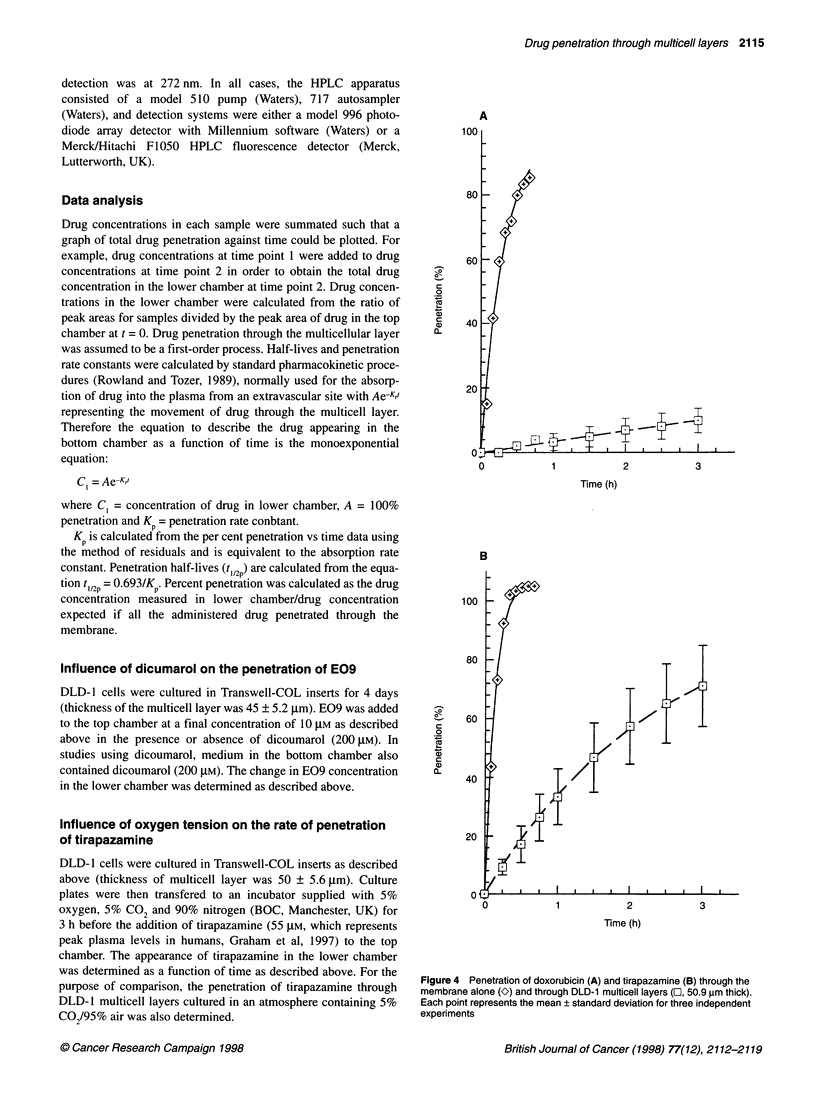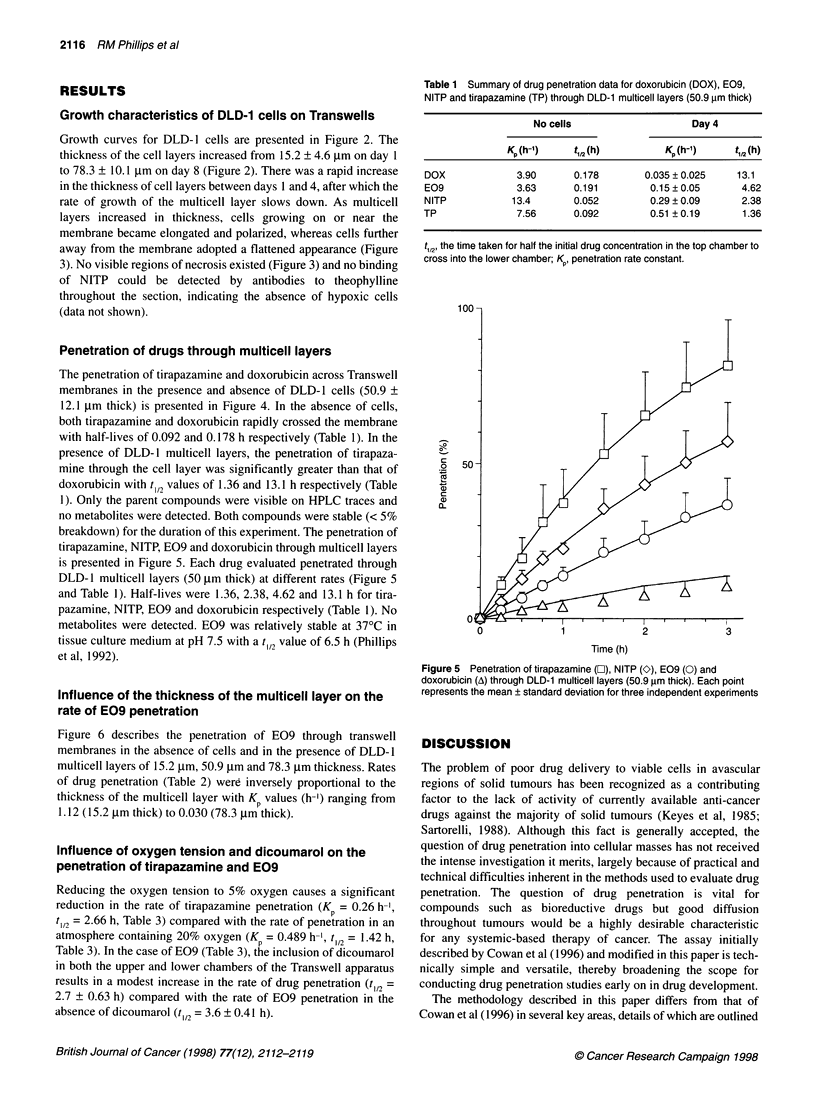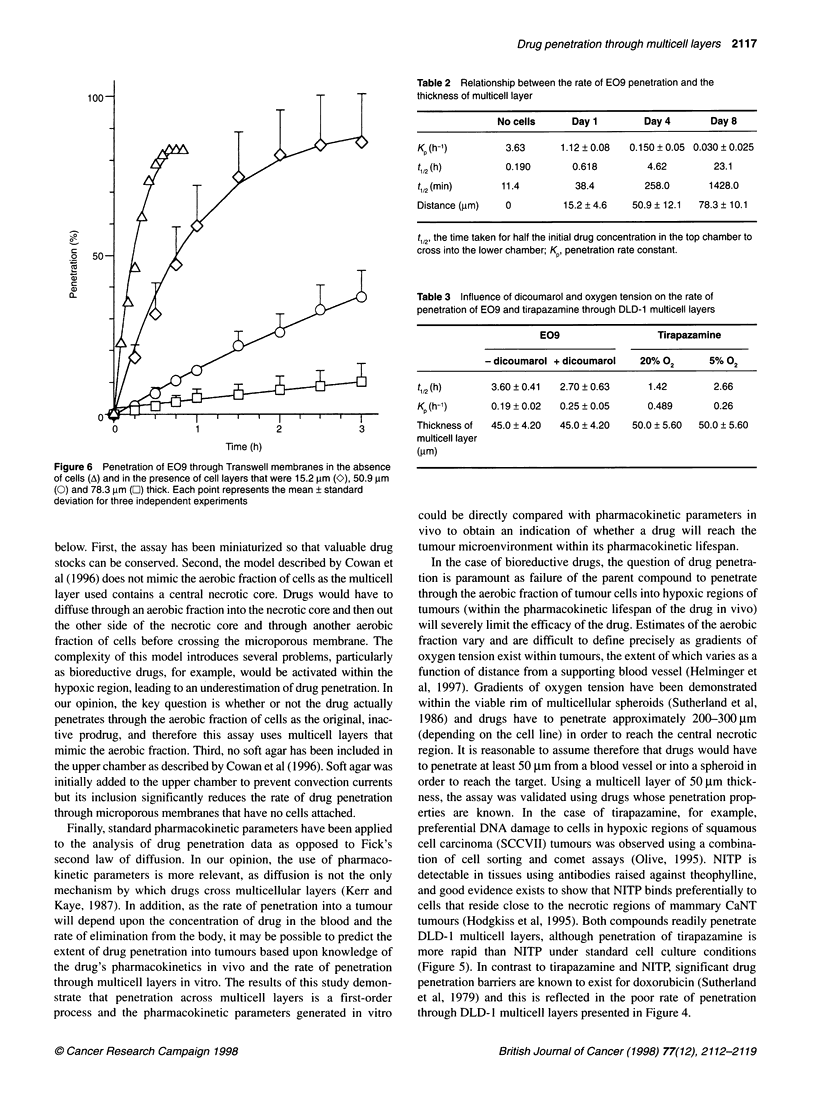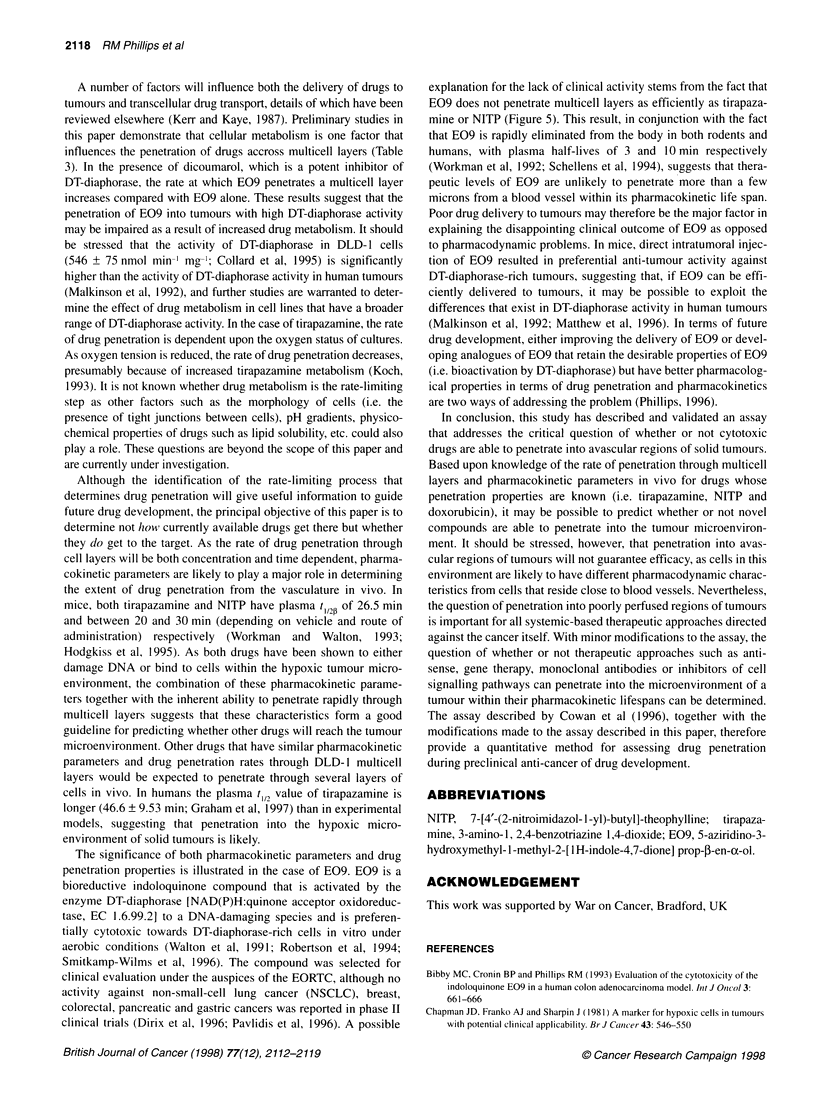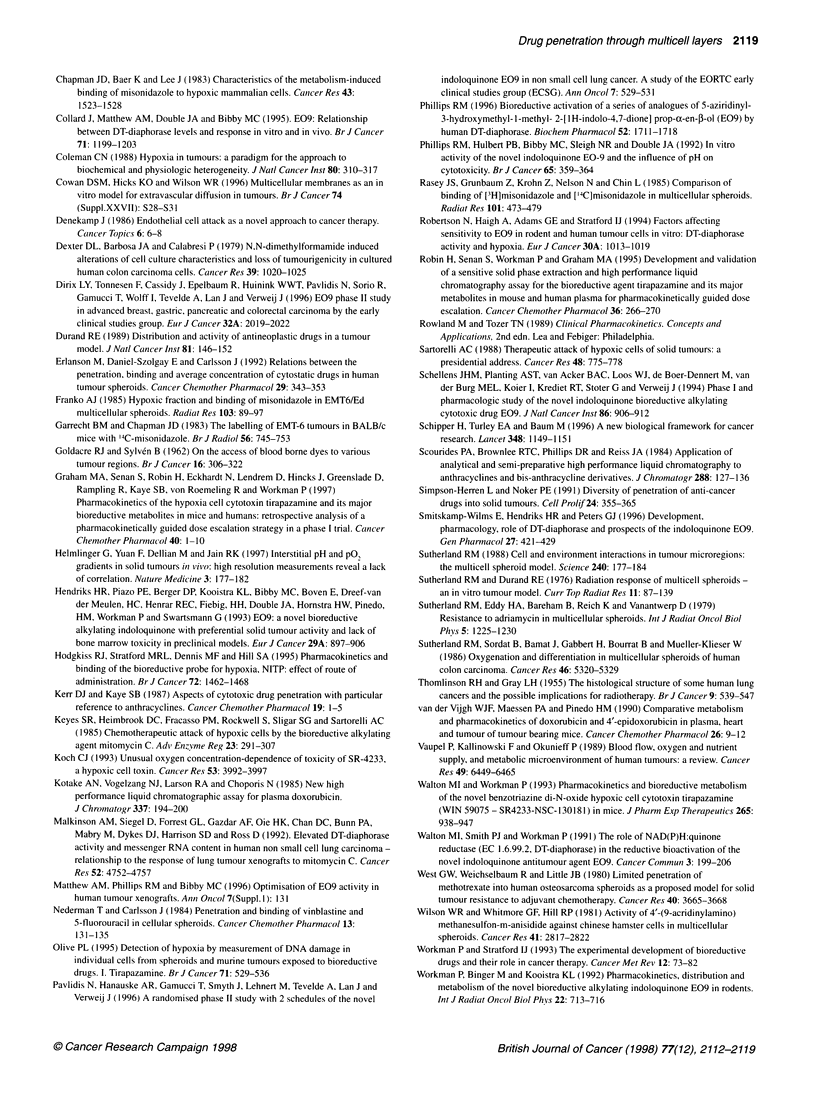# Evaluation of a novel in vitro assay for assessing drug penetration into avascular regions of tumours.

**DOI:** 10.1038/bjc.1998.355

**Published:** 1998-06

**Authors:** R. M. Phillips, P. M. Loadman, B. P. Cronin

**Affiliations:** Clinical Oncology Unit, University of Bradford, UK.

## Abstract

**Images:**


British Journal of Cancer (1998) 77(12), 2112–2119
© 1998 Cancer Research Campaign

### Evaluation of a novel in vitro assay for assessing drug penetration into avascular regions of tumours

**RM Phillips, PM Loadman and BP Cronin**

Clinical Oncology Unit, University of Bradford, Bradford BD7 1DP, UK

**Summary** The poor blood supply to solid tumours introduces many factors that affect the outcome of chemotherapy, one of which is the problem of drug delivery to poorly vascularized regions of tumours. Whereas poor drug penetration has been recognized as a contributing factor to the poor response of many solid tumours, the question of drug penetration through multicell layers has not been thoroughly addressed, largely because of restrictions imposed upon these studies by the requirement for either radiolabelled or naturally fluorescent compounds. The aim of this study is to describe modifications made to a recently published assay that broadens the scope for assessing drug penetration during the early stages of drug development and to characterize the ability of various drugs to penetrate multicell layers. DLD-1 human colon carcinoma cells were cultured on Transwell-COL plastic inserts placed into 24-well culture plates so that a top and bottom chamber were established, the two chambers being separated by a microporous membrane. Drugs were added to the top chamber at doses equivalent to peak plasma concentrations in vivo and the rate of appearance of drugs in the bottom chamber determined by high-performance liquid chromatography (HPLC). Both 3-amino-1,2,4-benzotriazine 1,4-dioxide (tirapazamine) and 7-[4′-(2-nitroimidazol-1-yl)-butyl]-theophylline (NITP) rapidly penetrated DLD-1 multicell layers (50.9 ± 12.1 μm thick) with $t_{1/2}$ values of 1.36 and 2.38 h respectively, whereas the rate of penetration of 5-aziridino-3-hydroxymethyl-1-methyl-2-[1H-indole-4,7-dione] prop-β-en-α-ol (EO9) and doxorubicin through multicell layers was significantly slower ($t_{1/2}$ = 4.62 and 13.1 h respectively). Inclusion of dicoumarol increases the rate of EO9 penetration, whereas reducing the oxygen tension to 5% causes a reduction in tirapazamine penetration through multicell layers, suggesting that the extent of drug metabolism is one factor that determines the rate at which drugs penetrate multicell layers. The fact that EO9 does not readily penetrate a multicell layer, in conjunction with its rapid elimination in vivo ($t_{1/2}$ < 10 min), suggests that EO9 is unlikely to penetrate more than a few μm from a blood vessel within its pharmacokinetic lifespan. These results suggest that the failure of EO9 in the clinic is due to a combination of poor drug penetration and rapid elimination in vivo.

**Keywords:** drug penetration; bioreductive drug; EO9; tirapazamine

The ultimate objective of any systemic therapy for cancer is to eradicate all tumour cells whether it is by direct cytotoxicity or by modifying the malignant phenotype (Schipper et al, 1996). In order to achieve this objective, it is essential that anti-cancer agents reach all clonogenic cells within the tumour at the concentrations that are required for a therapeutic effect. The blood supply to many solid tumours is known to be both inadequate and intermittent, resulting in regions of tumours that are chronically and transiently hypoxic (Coleman, 1988; Vaupel et al, 1989). Hypoxia by itself, or in conjunction with other features of the tumour microenvironment such as low extracellular pH and reduced cell proliferation rates, etc., can adversely affect the efficacy of both radiotherapy and chemotherapy (Thomlinson and Gray, 1955; Denekamp, 1986). In terms of chemotherapy, cytotoxic drugs have to diffuse from the vascular compartment and penetrate through several layers of cells in order to reach cells that reside some distance away from a blood vessel. Drug penetration barriers exist for a number of clinically used anti-cancer drugs, and the failure of drugs to penetrate throughout the tumour within their pharmacokinetic lifespan has been recognized as a contributing factor towards the poor response of many solid tumours to chemotherapy (Goldacre and Sylvén, 1962; Kerr and Kaye, 1987; Durand, 1989; Simpson-Herren and Noker, 1991). Multicellular spheroids (Sutherland and Durand, 1976; Sutherland, 1988) have been instrumental in the study of drug diffusion through multicell layers, and drug penetration barriers have been identified for drugs such as doxorubicin, methotrexate, m-AMSA, ara-C, vinblastine and vincristine (Sutherland et al, 1979; West et al, 1980; Wilson et al, 1981; Nederman and Carlsson, 1984; Erlanson et al, 1992).

Whereas the ability to penetrate several layers of cells is a desirable property of all systemic-based anti-cancer therapies, it is particularly relevant to a class of compounds known as bioreductive drugs. These compounds are designed to kill cells preferentially within the hypoxic tumour microenvironment (Sartorelli, 1988; Workman and Stratford, 1993) and the ideal bioreductive drug should be administered as an inactive prodrug that is only activated under low-oxygen conditions by one or two electron reductases. An essential prerequisite characteristic for an effective bioreductive drug is that the parent compound must diffuse through the aerobic fraction of cells in order to reach the target. With the exception of early studies using nitroimadazole-based radiosensitizers (Chapman et al, 1981, 1983; Garrecht and Chapman, 1983; Franko, 1985; Rasey et al, 1985), relatively few studies have focused on the ability of bioreductive drugs to penetrate through several layers of cells. This is due largely to the fact

*Received 20 June 1997*
*Accepted 5 December 1997*

*Correspondence to:* RM Phillips

**Figure 1** Schematic representation of the Transwell apparatus for assessing drug penetration that compounds have had to be either naturally fluorescent (such as doxorubicin) or radioactively labelled in order to detect penetration into cellular masses. In the early stages of drug development, however, radioactively labelled or fluorescent compounds are rarely available and, consequently, the question of drug penetration has not been thoroughly addressed for the majority of compounds.

Recently, an assay has been developed that has broadened the scope for studying drug penetration during the early stages of preclinical drug development (Cowan et al, 1996). The assay is based upon the ability of drugs to cross a microporous membrane that supports a multicell layer. Routine analytical techniques [e.g. high-performance liquid chromatography (HPLC)] can be used to measure drug (and/or metabolite) concentrations, making the study of drug penetration a feasible proposition for most laboratories involved in preclinical drug development. The principal objective of this study is to describe and characterize a modified version of the assay published by Cowan et al (1996) and to illustrate the potential significance of this assay by evaluating the ability of selected bioreductive and standard anti-cancer drugs to penetrate multicell layers. Particular attention has been paid to the bioreductive indoloquinone compound 5-aziridino-3-hydroxymethyl-1-methyl-2-[1H-indole-4,7-dione]prop-β-en-α-ol (EO9), which, despite promising activity in preclinical models (Hendriks et al, 1993), has proved disappointing in the clinic (Pavlidis et al, 1996). Studies demonstrating that spheroids are more resistant than monolayers to EO9 suggest that drug penetration barriers may exist (Bibby et al, 1993) and this study provides experimental evidence to support the hypothesis that the failure of EO9 in the clinic is due to poor penetration into tumours, in conjunction with rapid elimination kinetics in vivo.

### MATERIALS AND METHODS

### Test compounds

3-Amino-1,2,4-benzotriazine 1,4-dioxide (tirapazamine) and 7-[4′-(2-nitroimidazol-1-yl)butyl]-theophylline (NITP) were gifts from Sanofi Winthrop (USA) and Dr Richard Hodgkiss (Gray Laboratories, UK) respectively. EO9 was supplied by the New Drug Development Office of the EORTC (European Organization for the Research and Treatment of Cancer). Doxorubicin was purchased from Sigma (Sigma Aldrich, Poole, UK). Tirapazamine, NITP and EO9 were dissolved in DMSO and stored at – 20°C. Doxorubicin was dissolved in sterile water and stored at – 20°C. All chemicals used were of analytical grade (Sigma) and all solvents were HPLC grade (Fisher Scientific, Loughborough, UK).

### Cell culture conditions

DLD-1 human colon carcinoma cells (Dexter et al, 1979) were routinely maintained at 37°C as monolayer cultures in RPMI-1640 culture medium containing 25 mM HEPES buffer (Life Technologies, Paisley, UK) supplemented with 10% fetal calf serum (Life Technologies), sodium pyruvate (1 mM, Life Technologies,), penicillin–streptomycin (100 IU $ml^{-1}$:100 µg $ml^{-1}$, Life Technologies), L-glutamine (1 mM, Life Technologies).

### Growth characteristics of DLD-1 cells in Transwell culture vessels

A total of 2.5 × $10^5$ cells in 200 µl of RPMI-1640 culture medium was added to the top chamber of Transwell-COL plastic insert (Figure 1, Corning Costar, High Wycombe, UK). The top and bottom chambers were separated by a collagen-coated, microporous membrane (pore size 0.4 µm, diameter 6.5 mm, surface area 0.33 $cm^2$). Transwell vessels were incubated at 37°C for 3 h to allow cells to attach to the membrane before the addition of 2 ml of RPMI-1640 to the bottom chamber. Cells were incubated at 37°C in an atmosphere containing 5% $CO_2$ for up to 8 days with daily changes of medium in both upper and lower chambers. At various time points, Transwell inserts were removed, fixed in Bouin's fluid for 1 h and washed in 70% ethanol overnight. The membrane was detached from the plastic insert, embedded in paraffin wax and sectioned (5 µm) using a Leitz rotary microtome (Leica UK, Milton Keynes, UK). Sections were stained with haematoxylin and eosin according to standard protocols, and the average thickness of the multicell layer determined by a Seescan image analyser (Seescan, Cambridge, UK). Three separate Transwells per time point were sectioned and the thickness of each multicell layer was determined on five sections from each Transwell (20 measurements per section).

© Cancer Research Campaign 1998

**Figure 2** Growth of DLD-1 multicell layers on Transwell inserts. Each point represents the mean ± standard deviations for > 100 measurements on a total of five histological sections (three Transwells per time point)

**Figure 3** Histological sections through DLD-1 multicell layers on days 1 (**A**), 2 (**B**), 4 (**C**), 6 (**D**) and 8 (**E**) of the growth curve

### Drug penetration assay

Medium was removed from the top chamber of the Transwell and replaced with 100 µl of medium (phenol red-free RPMI-1640 medium supplemented with 10% fetal calf serum) containing drugs at doses that represent peak plasma drug concentrations in vivo (EO9 10 µM, tirapazamine 120 µM, NITP 100 µM and doxorubicin 10 µM; van der Vijgh et al, 1990; Bibby et al, 1993; Walton and Workman, 1993; Hodgkiss et al, 1995). In all cases, the final DMSO concentration was < 0.2%. The Transwell was then inserted into one well of a 24-well plate containing 600 µl of medium and incubated at 37°C. At various time intervals thereafter, 500 µl of medium was removed from the bottom chamber and added to 1 ml of acetonitrile, mixed and stored at – 20°C until required for analysis. The insert was then transferred to a different well on the culture plate containing 600 µl of fresh medium. This procedure was repeated throughout the duration of the experiment. At all stages of the process, medium in the bottom chamber was agitated using a small magnetic stirrer. In the case of EO9, drug penetration was assessed on days 1, 4 and 8 of the growth curve in order to determine the relationship between the thickness of the multicell layer and the rate of penetration. In the case of tirapazamine, NITP and doxorubicin, drug penetration was determined on day 4 of the growth curve. All experiments were repeated independently on three occasions. The stability of test compounds under the experimental conditions used (i.e. tissue culture medium at 37°C for 3 h) was determined according to the procedures described above.

### Sample analysis

Samples were evaporated down to 500 µl under vacuum using a RC10.10 centrifugal evaporator (Jouan, Ilkeston, UK). A further 100 µl of acetonitrile was added to each sample to make it more compatible with the mobile phases used. Following centrifugation (7000 ***g*** × 10 min), EO9 and doxorubicin concentrations in the supernatant were determined by HPLC according to previously published protocols (Scourides et al, 1984; Kotake et al, 1985; Phillips et al, 1992). In the case of tirapazamine, a modified version of the assay described by Robin et al (1995) was employed, brief details of which are described below. Samples were separated using a µBondapak (10 µm) phenyl column in a RCM 8 × 10 radial compression module (Waters, Watford, UK). The mobile phase was acetonitrile–water (11:89) pumped at a flow rate of 2.0 ml $min^{-1}$ and tirapazamine was detected at 266 nm. NITP was analysed using the same methodology as for tirapazamine except that the mobile phase was 30% acetonitrile–water and

© Cancer Research Campaign 1998

detection was at 272 nm. In all cases, the HPLC apparatus consisted of a model 510 pump (Waters), 717 autosampler (Waters), and detection systems were either a model 996 photodiode array detector with Millennium software (Waters) or a Merck/Hitachi F1050 HPLC fluorescence detector (Merck, Lutterworth, UK).

### Data analysis

Drug concentrations in each sample were summated such that a graph of total drug penetration against time could be plotted. For example, drug concentrations at time point 1 were added to drug concentrations at time point 2 in order to obtain the total drug concentration in the lower chamber at time point 2. Drug concentrations in the lower chamber were calculated from the ratio of peak areas for samples divided by the peak area of drug in the top chamber at $t = 0$. Drug penetration through the multicellular layer was assumed to be a first-order process. Half-lives and penetration rate constants were calculated by standard pharmacokinetic procedures (Rowland and Tozer, 1989), normally used for the absorption of drug into the plasma from an extravascular site with $Ae^{-K_pt}$ representing the movement of drug through the multicell layer. Therefore the equation to describe the drug appearing in the bottom chamber as a function of time is the monoexponential equation:

$$C_l = Ae^{-K_pt}$$

where $C_l$ = concentration of drug in lower chamber, $A$ = 100% penetration and $K_p$ = penetration rate conbtant.

$K_p$ is calculated from the per cent penetration vs time data using the method of residuals and is equivalent to the absorption rate constant. Penetration half-lives ($t_{1/2p}$) are calculated from the equation $t_{1/2p} = 0.693/K_p$. Percent penetration was calculated as the drug concentration measured in lower chamber/drug concentration expected if all the administered drug penetrated through the membrane.

### Influence of dicumarol on the penetration of EO9

DLD-1 cells were cultured in Transwell-COL inserts for 4 days (thickness of the multicell layer was 45 ± 5.2 μm). EO9 was added to the top chamber at a final concentration of 10 μM as described above in the presence or absence of dicoumarol (200 μM). In studies using dicoumarol, medium in the bottom chamber also contained dicoumarol (200 μM). The change in EO9 concentration in the lower chamber was determined as described above.

### Influence of oxygen tension on the rate of penetration of tirapazamine

DLD-1 cells were cultured in Transwell-COL inserts as described above (thickness of multicell layer was 50 ± 5.6 μm). Culture plates were then transfered to an incubator supplied with 5% oxygen, 5% $CO_2$ and 90% nitrogen (BOC, Manchester, UK) for 3 h before the addition of tirapazamine (55 μM, which represents peak plasma levels in humans, Graham et al, 1997) to the top chamber. The appearance of tirapazamine in the lower chamber was determined as a function of time as described above. For the purpose of comparison, the penetration of tirapazamine through DLD-1 multicell layers cultured in an atmosphere containing 5% $CO_2$/95% air was also determined.

**Figure 4** Penetration of doxorubicin (**A**) and tirapazamine (**B**) through the membrane alone (◇) and through DLD-1 multicell layers (□, 50.9 μm thick). Each point represents the mean ± standard deviation for three independent experiments

### RESULTS

### Growth characteristics of DLD-1 cells on Transwells

Growth curves for DLD-1 cells are presented in Figure 2. The thickness of the cell layers increased from 15.2 ± 4.6 μm on day 1 to 78.3 ± 10.1 μm on day 8 (Figure 2). There was a rapid increase in the thickness of cell layers between days 1 and 4, after which the rate of growth of the multicell layer slows down. As multicell layers increased in thickness, cells growing on or near the membrane became elongated and polarized, whereas cells further away from the membrane adopted a flattened appearance (Figure 3). No visible regions of necrosis existed (Figure 3) and no binding of NITP could be detected by antibodies to theophylline throughout the section, indicating the absence of hypoxic cells (data not shown).

### Penetration of drugs through multicell layers

The penetration of tirapazamine and doxorubicin across Transwell membranes in the presence and absence of DLD-1 cells (50.9 ± 12.1 μm thick) is presented in Figure 4. In the absence of cells, both tirapazamine and doxorubicin rapidly crossed the membrane with half-lives of 0.092 and 0.178 h respectively (Table 1). In the presence of DLD-1 multicell layers, the penetration of tirapazamine through the cell layer was significantly greater than that of doxorubicin with $t_{1/2}$ values of 1.36 and 13.1 h respectively (Table 1). Only the parent compounds were visible on HPLC traces and no metabolites were detected. Both compounds were stable (< 5% breakdown) for the duration of this experiment. The penetration of tirapazamine, NITP, EO9 and doxorubicin through multicell layers is presented in Figure 5. Each drug evaluated penetrated through DLD-1 multicell layers (50 μm thick) at different rates (Figure 5 and Table 1). Half-lives were 1.36, 2.38, 4.62 and 13.1 h for tirapazamine, NITP, EO9 and doxorubicin respectively (Table 1). No metabolites were detected. EO9 was relatively stable at 37°C in tissue culture medium at pH 7.5 with a $t_{1/2}$ value of 6.5 h (Phillips et al, 1992).

### Influence of the thickness of the multicell layer on the rate of EO9 penetration

Figure 6 describes the penetration of EO9 through transwell membranes in the absence of cells and in the presence of DLD-1 multicell layers of 15.2 μm, 50.9 μm and 78.3 μm thickness. Rates of drug penetration (Table 2) were inversely proportional to the thickness of the multicell layer with $K_p$ values ($h^{-1}$) ranging from 1.12 (15.2 μm thick) to 0.030 (78.3 μm thick).

### Influence of oxygen tension and dicoumarol on the penetration of tirapazamine and EO9

Reducing the oxygen tension to 5% oxygen causes a significant reduction in the rate of tirapazamine penetration ($K_p$ = 0.26 $h^{-1}$, $t_{1/2}$ = 2.66 h, Table 3) compared with the rate of penetration in an atmosphere containing 20% oxygen ($K_p$ = 0.489 $h^{-1}$, $t_{1/2}$ = 1.42 h, Table 3). In the case of EO9 (Table 3), the inclusion of dicoumarol in both the upper and lower chambers of the Transwell apparatus results in a modest increase in the rate of drug penetration ($t_{1/2}$ = 2.7 ± 0.63 h) compared with the rate of EO9 penetration in the absence of dicoumarol ($t_{1/2}$ = 3.6 ± 0.41 h).

**Table 1** Summary of drug penetration data for doxorubicin (DOX), EO9, NITP and tirapazamine (TP) through DLD-1 multicell layers (50.9 μm thick)

| | No cells | | Day 4 | |
|---|---|---|---|---|
| | $K_p$ ($h^{-1}$) | $t_{1/2}$ (h) | $K_p$ ($h^{-1}$) | $t_{1/2}$ (h) |
| DOX | 3.90 | 0.178 | 0.035 ± 0.025 | 13.1 |
| EO9 | 3.63 | 0.191 | 0.15 ± 0.05 | 4.62 |
| NITP | 13.4 | 0.052 | 0.29 ± 0.09 | 2.38 |
| TP | 7.56 | 0.092 | 0.51 ± 0.19 | 1.36 |

$t_{1/2}$, the time taken for half the initial drug concentration in the top chamber to cross into the lower chamber; $K_p$, penetration rate constant.

**Figure 5** Penetration of tirapazamine (□), NITP (◇), EO9 (○) and doxorubicin (Δ) through DLD-1 multicell layers (50.9 μm thick). Each point represents the mean ± standard deviation for three independent experiments

### DISCUSSION

The problem of poor drug delivery to viable cells in avascular regions of solid tumours has been recognized as a contributing factor to the lack of activity of currently available anti-cancer drugs against the majority of solid tumours (Keyes et al, 1985; Sartorelli, 1988). Although this fact is generally accepted, the question of drug penetration into cellular masses has not received the intense investigation it merits, largely because of practical and technical difficulties inherent in the methods used to evaluate drug penetration. The question of drug penetration is vital for compounds such as bioreductive drugs but good diffusion throughout tumours would be a highly desirable characteristic for any systemic-based therapy of cancer. The assay initially described by Cowan et al (1996) and modified in this paper is technically simple and versatile, thereby broadening the scope for conducting drug penetration studies early on in drug development.

The methodology described in this paper differs from that of Cowan et al (1996) in several key areas, details of which are outlined

© Cancer Research Campaign 1998

**Figure 6** Penetration of EO9 through Transwell membranes in the absence of cells (Δ) and in the presence of cell layers that were 15.2 μm (◇), 50.9 μm (○) and 78.3 μm (□) thick. Each point represents the mean ± standard deviation for three independent experiments below. First, the assay has been miniaturized so that valuable drug stocks can be conserved. Second, the model described by Cowan et al (1996) does not mimic the aerobic fraction of cells as the multicell layer used contains a central necrotic core. Drugs would have to diffuse through an aerobic fraction into the necrotic core and then out the other side of the necrotic core and through another aerobic fraction of cells before crossing the microporous membrane. The complexity of this model introduces several problems, particularly as bioreductive drugs, for example, would be activated within the hypoxic region, leading to an underestimation of drug penetration. In our opinion, the key question is whether or not the drug actually penetrates through the aerobic fraction of cells as the original, inactive prodrug, and therefore this assay uses multicell layers that mimic the aerobic fraction. Third, no soft agar has been included in the upper chamber as described by Cowan et al (1996). Soft agar was initially added to the upper chamber to prevent convection currents but its inclusion significantly reduces the rate of drug penetration through microporous membranes that have no cells attached.

Finally, standard pharmacokinetic parameters have been applied to the analysis of drug penetration data as opposed to Fick's second law of diffusion. In our opinion, the use of pharmacokinetic parameters is more relevant, as diffusion is not the only mechanism by which drugs cross multicellular layers (Kerr and Kaye, 1987). In addition, as the rate of penetration into a tumour will depend upon the concentration of drug in the blood and the rate of elimination from the body, it may be possible to predict the extent of drug penetration into tumours based upon knowledge of the drug's pharmacokinetics in vivo and the rate of penetration through multicell layers in vitro. The results of this study demonstrate that penetration across multicell layers is a first-order process and the pharmacokinetic parameters generated in vitro

**Table 2** Relationship between the rate of EO9 penetration and the thickness of multicell layer

| | No cells | Day 1 | Day 4 | Day 8 |
|---|---|---|---|---|
| $K_p$ (h$^{-1}$) | 3.63 | 1.12 ± 0.08 | 0.150 ± 0.05 | 0.030 ± 0.025 |
| $t_{1/2}$ (h) | 0.190 | 0.618 | 4.62 | 23.1 |
| $t_{1/2}$ (min) | 11.4 | 38.4 | 258.0 | 1428.0 |
| Distance (μm) | 0 | 15.2 ± 4.6 | 50.9 ± 12.1 | 78.3 ± 10.1 |

$t_{1/2}$, the time taken for half the initial drug concentration in the top chamber to cross into the lower chamber; $K_p$, penetration rate constant.

**Table 3** Influence of dicoumarol and oxygen tension on the rate of penetration of EO9 and tirapazamine through DLD-1 multicell layers

| | EO9 | | Tirapazamine | |
|---|---|---|---|---|
| | – dicoumarol | + dicoumarol | 20% $O_2$ | 5% $O_2$ |
| $t_{1/2}$ (h) | 3.60 ± 0.41 | 2.70 ± 0.63 | 1.42 | 2.66 |
| $K_p$ (h$^{-1}$) | 0.19 ± 0.02 | 0.25 ± 0.05 | 0.489 | 0.26 |
| Thickness of multicell layer (μm) | 45.0 ± 4.20 | 45.0 ± 4.20 | 50.0 ± 5.60 | 50.0 ± 5.60 |

could be directly compared with pharmacokinetic parameters in vivo to obtain an indication of whether a drug will reach the tumour microenvironment within its pharmacokinetic lifespan.

In the case of bioreductive drugs, the question of drug penetration is paramount as failure of the parent compound to penetrate through the aerobic fraction of tumour cells into hypoxic regions of tumours (within the pharmacokinetic lifespan of the drug in vivo) will severely limit the efficacy of the drug. Estimates of the aerobic fraction vary and are difficult to define precisely as gradients of oxygen tension exist within tumours, the extent of which varies as a function of distance from a supporting blood vessel (Helminger et al, 1997). Gradients of oxygen tension have been demonstrated within the viable rim of multicellular spheroids (Sutherland et al, 1986) and drugs have to penetrate approximately 200–300 μm (depending on the cell line) in order to reach the central necrotic region. It is reasonable to assume therefore that drugs would have to penetrate at least 50 μm from a blood vessel or into a spheroid in order to reach the target. Using a multicell layer of 50 μm thickness, the assay was validated using drugs whose penetration properties are known. In the case of tirapazamine, for example, preferential DNA damage to cells in hypoxic regions of squamous cell carcinoma (SCCVII) tumours was observed using a combination of cell sorting and comet assays (Olive, 1995). NITP is detectable in tissues using antibodies raised against theophylline, and good evidence exists to show that NITP binds preferentially to cells that reside close to the necrotic regions of mammary CaNT tumours (Hodgkiss et al, 1995). Both compounds readily penetrate DLD-1 multicell layers, although penetration of tirapazamine is more rapid than NITP under standard cell culture conditions (Figure 5). In contrast to tirapazamine and NITP, significant drug penetration barriers are known to exist for doxorubicin (Sutherland et al, 1979) and this is reflected in the poor rate of penetration through DLD-1 multicell layers presented in Figure 4.

A number of factors will influence both the delivery of drugs to tumours and transcellular drug transport, details of which have been reviewed elsewhere (Kerr and Kaye, 1987). Preliminary studies in this paper demonstrate that cellular metabolism is one factor that influences the penetration of drugs accross multicell layers (Table 3). In the presence of dicoumarol, which is a potent inhibitor of DT-diaphorase, the rate at which EO9 penetrates a multicell layer increases compared with EO9 alone. These results suggest that the penetration of EO9 into tumours with high DT-diaphorase activity may be impaired as a result of increased drug metabolism. It should be stressed that the activity of DT-diaphorase in DLD-1 cells (546 ± 75 nmol $min^{-1}$ $mg^{-1}$; Collard et al, 1995) is significantly higher than the activity of DT-diaphorase activity in human tumours (Malkinson et al, 1992), and further studies are warranted to determine the effect of drug metabolism in cell lines that have a broader range of DT-diaphorase activity. In the case of tirapazamine, the rate of drug penetration is dependent upon the oxygen status of cultures. As oxygen tension is reduced, the rate of drug penetration decreases, presumably because of increased tirapazamine metabolism (Koch, 1993). It is not known whether drug metabolism is the rate-limiting step as other factors such as the morphology of cells (i.e. the presence of tight junctions between cells), pH gradients, physicochemical properties of drugs such as lipid solubility, etc. could also play a role. These questions are beyond the scope of this paper and are currently under investigation.

Although the identification of the rate-limiting process that determines drug penetration will give useful information to guide future drug development, the principal objective of this paper is to determine not *how* currently available drugs get there but whether they *do* get to the target. As the rate of drug penetration through cell layers will be both concentration and time dependent, pharmacokinetic parameters are likely to play a major role in determining the extent of drug penetration from the vasculature in vivo. In mice, both tirapazamine and NITP have plasma $t_{1/2\beta}$ of 26.5 min and between 20 and 30 min (depending on vehicle and route of administration) respectively (Workman and Walton, 1993; Hodgkiss et al, 1995). As both drugs have been shown to either damage DNA or bind to cells within the hypoxic tumour microenvironment, the combination of these pharmacokinetic parameters together with the inherent ability to penetrate rapidly through multicell layers suggests that these characteristics form a good guideline for predicting whether other drugs will reach the tumour microenvironment. Other drugs that have similar pharmacokinetic parameters and drug penetration rates through DLD-1 multicell layers would be expected to penetrate through several layers of cells in vivo. In humans the plasma $t_{1/2}$ value of tirapazamine is longer (46.6 ± 9.53 min; Graham et al, 1997) than in experimental models, suggesting that penetration into the hypoxic microenvironment of solid tumours is likely.

The significance of both pharmacokinetic parameters and drug penetration properties is illustrated in the case of EO9. EO9 is a bioreductive indoloquinone compound that is activated by the enzyme DT-diaphorase [NAD(P)H:quinone acceptor oxidoreductase, EC 1.6.99.2] to a DNA-damaging species and is preferentially cytotoxic towards DT-diaphorase-rich cells in vitro under aerobic conditions (Walton et al, 1991; Robertson et al, 1994; Smitkamp-Wilms et al, 1996). The compound was selected for clinical evaluation under the auspices of the EORTC, although no activity against non-small-cell lung cancer (NSCLC), breast, colorectal, pancreatic and gastric cancers was reported in phase II clinical trials (Dirix et al, 1996; Pavlidis et al, 1996). A possible explanation for the lack of clinical activity stems from the fact that EO9 does not penetrate multicell layers as efficiently as tirapazamine or NITP (Figure 5). This result, in conjunction with the fact that EO9 is rapidly eliminated from the body in both rodents and humans, with plasma half-lives of 3 and 10 min respectively (Workman et al, 1992; Schellens et al, 1994), suggests that therapeutic levels of EO9 are unlikely to penetrate more than a few microns from a blood vessel within its pharmacokinetic life span. Poor drug delivery to tumours may therefore be the major factor in explaining the disappointing clinical outcome of EO9 as opposed to pharmacodynamic problems. In mice, direct intratumoral injection of EO9 resulted in preferential anti-tumour activity against DT-diaphorase-rich tumours, suggesting that, if EO9 can be efficiently delivered to tumours, it may be possible to exploit the differences that exist in DT-diaphorase activity in human tumours (Malkinson et al, 1992; Matthew et al, 1996). In terms of future drug development, either improving the delivery of EO9 or developing analogues of EO9 that retain the desirable properties of EO9 (i.e. bioactivation by DT-diaphorase) but have better pharmacological properties in terms of drug penetration and pharmacokinetics are two ways of addressing the problem (Phillips, 1996).

In conclusion, this study has described and validated an assay that addresses the critical question of whether or not cytotoxic drugs are able to penetrate into avascular regions of solid tumours. Based upon knowledge of the rate of penetration through multicell layers and pharmacokinetic parameters in vivo for drugs whose penetration properties are known (i.e. tirapazamine, NITP and doxorubicin), it may be possible to predict whether or not novel compounds are able to penetrate into the tumour microenvironment. It should be stressed, however, that penetration into avascular regions of tumours will not guarantee efficacy, as cells in this environment are likely to have different pharmacodynamic characteristics from cells that reside close to blood vessels. Nevertheless, the question of penetration into poorly perfused regions of tumours is important for all systemic-based therapeutic approaches directed against the cancer itself. With minor modifications to the assay, the question of whether or not therapeutic approaches such as antisense, gene therapy, monoclonal antibodies or inhibitors of cell signalling pathways can penetrate into the microenvironment of a tumour within their pharmacokinetic lifespans can be determined. The assay described by Cowan et al (1996), together with the modifications made to the assay described in this paper, therefore provide a quantitative method for assessing drug penetration during preclinical anti-cancer of drug development.

### ABBREVIATIONS

NITP, 7-[4′-(2-nitroimidazol-1-yl)-butyl]-theophylline; tirapazamine, 3-amino-1, 2,4-benzotriazine 1,4-dioxide; EO9, 5-aziridino-3-hydroxymethyl-1-methyl-2-[1H-indole-4,7-dione] prop-β-en-α-ol.

### ACKNOWLEDGEMENT

This work was supported by War on Cancer, Bradford, UK

### REFERENCES

Bibby MC, Cronin BP and Phillips RM (1993) Evaluation of the cytotoxicity of the indoloquinone EO9 in a human colon adenocarcinoma model. *Int J Oncol* **3**: 661–666

Chapman JD, Franko AJ and Sharpin J (1981) A marker for hypoxic cells in tumours with potential clinical applicability. *Br J Cancer* **43**: 546–550

© Cancer Research Campaign 1998

Chapman JD, Baer K and Lee J (1983) Characteristics of the metabolism-induced binding of misonidazole to hypoxic mammalian cells. *Cancer Res* **43**: 1523–1528

Collard J, Matthew AM, Double JA and Bibby MC (1995). EO9: Relationship between DT-diaphorase levels and response in vitro and in vivo. *Br J Cancer* **71**: 1199–1203

Coleman CN (1988) Hypoxia in tumours: a paradigm for the approach to biochemical and physiologic heterogeneity. *J Natl Cancer Inst* **80**: 310–317

Cowan DSM, Hicks KO and Wilson WR (1996) Multicellular membranes as an in vitro model for extravascular diffusion in tumours. *Br J Cancer* **74** (Suppl.XXVII): S28–S31

Denekamp J (1986) Endothelial cell attack as a novel approach to cancer therapy. *Cancer Topics* **6**: 6–8

Dexter DL, Barbosa JA and Calabresi P (1979) N,N-dimethylformamide induced alterations of cell culture characteristics and loss of tumourigenicity in cultured human colon carcinoma cells. *Cancer Res* **39**: 1020–1025

Dirix LY, Tonnesen F, Cassidy J, Epelbaum R, Huinink WWT, Pavlidis N, Sorio R, Gamucci T, Wolff I, Tevelde A, Lan J and Verweij J (1996) EO9 phase II study in advanced breast, gastric, pancreatic and colorectal carcinoma by the early clinical studies group. *Eur J Cancer* **32A**: 2019–2022

Durand RE (1989) Distribution and activity of antineoplastic drugs in a tumour model. *J Natl Cancer Inst* **81**: 146–152

Erlanson M, Daniel-Szolgay E and Carlsson J (1992) Relations between the penetration, binding and average concentration of cytostatic drugs in human tumour spheroids. *Cancer Chemother Pharmacol* **29**: 343–353

Franko AJ (1985) Hypoxic fraction and binding of misonidazole in EMT6/Ed multicellular spheroids. *Radiat Res* **103**: 89–97

Garrecht BM and Chapman JD (1983) The labelling of EMT-6 tumours in BALB/c mice with $^{14}$C-misonidazole. *Br J Radiol* **56**: 745–753

Goldacre RJ and Sylvén B (1962) On the access of blood borne dyes to various tumour regions. *Br J Cancer* **16**: 306–322

Graham MA, Senan S, Robin H, Eckhardt N, Lendrem D, Hincks J, Greenslade D, Rampling R, Kaye SB, von Roemeling R and Workman P (1997) Pharmacokinetics of the hypoxia cell cytotoxin tirapazamine and its major bioreductive metabolites in mice and humans: retrospective analysis of a pharmacokinetically guided dose escalation strategy in a phase I trial. *Cancer Chemother Pharmacol* **40**: 1–10

Helmlinger G, Yuan F, Dellian M and Jain RK (1997) Interstitial pH and $pO_2$ gradients in solid tumours *in vivo*: high resolution measurements reveal a lack of correlation. *Nature Medicine* **3**: 177–182

Hendriks HR, Piazo PE, Berger DP, Kooistra KL, Bibby MC, Boven E, Dreef-van der Meulen, HC, Henrar REC, Fiebig, HH, Double JA, Hornstra HW, Pinedo, HM, Workman P and Swartsmann G (1993) EO9: a novel bioreductive alkylating indoloquinone with preferential solid tumour activity and lack of bone marrow toxicity in preclinical models. *Eur J Cancer* **29A**: 897–906

Hodgkiss RJ, Stratford MRL, Dennis MF and Hill SA (1995) Pharmacokinetics and binding of the bioreductive probe for hypoxia, NITP: effect of route of administration. *Br J Cancer* **72**: 1462–1468

Kerr DJ and Kaye SB (1987) Aspects of cytotoxic drug penetration with particular reference to anthracyclines. *Cancer Chemother Pharmacol* **19**: 1–5

Keyes SR, Heimbrook DC, Fracasso PM, Rockwell S, Sligar SG and Sartorelli AC (1985) Chemotherapeutic attack of hypoxic cells by the bioreductive alkylating agent mitomycin C. *Adv Enzyme Reg* **23**: 291–307

Koch CJ (1993) Unusual oxygen concentration-dependence of toxicity of SR-4233, a hypoxic cell toxin. *Cancer Res* **53**: 3992–3997

Kotake AN, Vogelzang NJ, Larson RA and Choporis N (1985) New high performance liquid chromatographic assay for plasma doxorubicin. *J Chromatogr* **337**: 194–200

Malkinson AM, Siegel D, Forrest GL, Gazdar AF, Oie HK, Chan DC, Bunn PA, Mabry M, Dykes DJ, Harrison SD and Ross D (1992). Elevated DT-diaphorase activity and messenger RNA content in human non small cell lung carcinoma – relationship to the response of lung tumour xenografts to mitomycin C. *Cancer Res* **52**: 4752–4757

Matthew AM, Phillips RM and Bibby MC (1996) Optimisation of EO9 activity in human tumour xenografts. *Ann Oncol* **7**(Suppl.1): 131

Nederman T and Carlsson J (1984) Penetration and binding of vinblastine and 5-fluorouracil in cellular spheroids. *Cancer Chemother Pharmacol* **13**: 131–135

Olive PL (1995) Detection of hypoxia by measurement of DNA damage in individual cells from spheroids and murine tumours exposed to bioreductive drugs. I. Tirapazamine. *Br J Cancer* **71**: 529–536

Pavlidis N, Hanauske AR, Gamucci T, Smyth J, Lehnert M, Tevelde A, Lan J and Verweij J (1996) A randomised phase II study with 2 schedules of the novel indoloquinone EO9 in non small cell lung cancer. A study of the EORTC early clinical studies group (ECSG). *Ann Oncol* **7**: 529–531

Phillips RM (1996) Bioreductive activation of a series of analogues of 5-aziridinyl-3-hydroxymethyl-1-methyl- 2-[1H-indolo-4,7-dione] prop-α-en-β-ol (EO9) by human DT-diaphorase. *Biochem Pharmacol* **52**: 1711–1718

Phillips RM, Hulbert PB, Bibby MC, Sleigh NR and Double JA (1992) In vitro activity of the novel indoloquinone EO-9 and the influence of pH on cytotoxicity. *Br J Cancer* **65**: 359–364

Rasey JS, Grunbaum Z, Krohn Z, Nelson N and Chin L (1985) Comparison of binding of [$^{3}$H]misonidazole and [$^{14}$C]misonidazole in multicellular spheroids. *Radiat Res* **101**: 473–479

Robertson N, Haigh A, Adams GE and Stratford IJ (1994) Factors affecting sensitivity to EO9 in rodent and human tumour cells in vitro: DT-diaphorase activity and hypoxia. *Eur J Cancer* **30A**: 1013–1019

Robin H, Senan S, Workman P and Graham MA (1995) Development and validation of a sensitive solid phase extraction and high performance liquid chromatography assay for the bioreductive agent tirapazamine and its major metabolites in mouse and human plasma for pharmacokinetically guided dose escalation. *Cancer Chemother Pharmacol* **36**: 266–270

Rowland M and Tozer TN (1989) *Clinical Pharmacokinetics. Concepts and Applications,* 2nd edn. Lea and Febiger: Philadelphia.

Sartorelli AC (1988) Therapeutic attack of hypoxic cells of solid tumours: a presidential address. *Cancer Res* **48**: 775–778

Schellens JHM, Planting AST, van Acker BAC, Loos WJ, de Boer-Dennert M, van der Burg MEL, Koier I, Krediet RT, Stoter G and Verweij J (1994) Phase I and pharmacologic study of the novel indoloquinone bioreductive alkylating cytotoxic drug EO9. *J Natl Cancer Inst* **86**: 906–912

Schipper H, Turley EA and Baum M (1996) A new biological framework for cancer research. *Lancet* **348**: 1149–1151

Scourides PA, Brownlee RTC, Phillips DR and Reiss JA (1984) Application of analytical and semi-preparative high performance liquid chromatography to anthracyclines and bis-anthracycline derivatives. *J Chromatogr* **288**: 127–136

Simpson-Herren L and Noker PE (1991) Diversity of penetration of anti-cancer drugs into solid tumours. *Cell Prolif* **24**: 355–365

Smitskamp-Wilms E, Hendriks HR and Peters GJ (1996) Development, pharmacology, role of DT-diaphorase and prospects of the indoloquinone EO9. *Gen Pharmacol* **27**: 421–429

Sutherland RM (1988) Cell and environment interactions in tumour microregions: the multicell spheroid model. *Science* **240**: 177–184

Sutherland RM and Durand RE (1976) Radiation response of multicell spheroids – an in vitro tumour model. *Curr Top Radiat Res* **11**: 87–139

Sutherland RM, Eddy HA, Bareham B, Reich K and Vanantwerp D (1979) Resistance to adriamycin in multicellular spheroids. *Int J Radiat Oncol Biol Phys* **5**: 1225–1230

Sutherland RM, Sordat B, Bamat J, Gabbert H, Bourrat B and Mueller-Klieser W (1986) Oxygenation and differentiation in multicellular spheroids of human colon carcinoma. *Cancer Res* **46**: 5320–5329

Thomlinson RH and Gray LH (1955) The histological structure of some human lung cancers and the possible implications for radiotherapy. *Br J Cancer* **9**: 539–547

van der Vijgh WJF, Maessen PA and Pinedo HM (1990) Comparative metabolism and pharmacokinetics of doxorubicin and 4′-epidoxorubicin in plasma, heart and tumour of tumour bearing mice. *Cancer Chemother Pharmacol* **26**: 9–12

Vaupel P, Kallinowski F and Okunieff P (1989) Blood flow, oxygen and nutrient supply, and metabolic microenvironment of human tumours: a review. *Cancer Res* **49**: 6449–6465

Walton MI and Workman P (1993) Pharmacokinetics and bioreductive metabolism of the novel benzotriazine di-N-oxide hypoxic cell cytotoxin tirapazamine (WIN 59075 – SR4233-NSC-130181) in mice. *J Pharm Exp Therapeutics* **265**: 938–947

Walton MI, Smith PJ and Workman P (1991) The role of NAD(P)H:quinone reductase (EC 1.6.99.2, DT-diaphorase) in the reductive bioactivation of the novel indoloquinone antitumour agent EO9. *Cancer Commun* **3**: 199–206

West GW, Weichselbaum R and Little JB (1980) Limited penetration of methotrexate into human osteosarcoma spheroids as a proposed model for solid tumour resistance to adjuvant chemotherapy. *Cancer Res* **40**: 3665–3668

Wilson WR and Whitmore GF, Hill RP (1981) Activity of 4′-(9-acridinylamino) methanesulfon-m-anisidide against chinese hamster cells in multicellular spheroids. *Cancer Res* **41**: 2817–2822

Workman P and Stratford IJ (1993) The experimental development of bioreductive drugs and their role in cancer therapy. *Cancer Met Rev* **12**: 73–82

Workman P, Binger M and Kooistra KL (1992) Pharmacokinetics, distribution and metabolism of the novel bioreductive alkylating indoloquinone EO9 in rodents. *Int J Radiat Oncol Biol Phys* **22**: 713–716

© Cancer Research Campaign 1998